# Editorial: Alternative and novel livestock feed: reducing environmental impact

**DOI:** 10.3389/fvets.2024.1441905

**Published:** 2024-07-18

**Authors:** Alessandro Vastolo, Francesco Serrapica, Damiano Cavallini, Isa Fusaro, Alberto Stanislo Atzori, Massimo Todaro

**Affiliations:** ^1^Department of Veterinary Medicine and Animal Production, University of Napoli Federico II, Naples, Italy; ^2^Department of Agriculture, University of Napoli Federico II, Portici, Italy; ^3^Department of Veterinary Medical Sciences (DIMEVET), Alma Mater Studiorum - University of Bologna, Bologna, Italy; ^4^Department of Veterinary Medicine, University of Teramo, Teramo, Italy; ^5^Department of Agriculture, University of Sassari, Sassari, Italy; ^6^Department of Agricultural, Food and Forest Science (SAAF), University of Palermo, Palermo, Italy

**Keywords:** alternative feed, additive, methane, flavonoid, ruminant, pig, poultry

## Introduction

The Food and Agriculture Organization (FAO) has stated that livestock production systems are responsible for 14.5% of anthropogenic greenhouse gas (GHG) emissions, which mainly result from enteric fermentation. Depending on the production system used, feed accounts for 55%−75% of the climate change impact (1). Cultivation and processing, transport and land-use change are the main global sources of GHG emissions from animal feed production. In addition, enteric methane emissions from ruminants and monogastric animals contribute significantly to the environmental footprint of agriculture (2). Methane and ammonia emissions also represent a significant loss of feed energy. The use of alternative feeds and additives could improve total digestibility and have significantly greater potential to improve animal performance and reduce emissions. However, the feasibility of using alternative feeds depends on the nutritional value of novel ingredients, animal production responses and feed costs compared to the conventional feeds.

In this Research Topic, 17 research article and 1 review were collected on the use of alternative feeds in animal nutrition.

## Alternative feed in ruminants and pigs

In ruminant and monogastric animals, the main aim of testing alternative feeds is to reduce the environmental impact of livestock animals and to reduce the feed costs of conventional feeds in order to improve animal performance. In the editorial, several authors approach the use of different alternative feeds by in *vitro* e and *in vivo* experiments. Li et al. investigated the effects of rice straw different particle sizes on the rumen protozoan count, nutrient disappearance rate, rumen fermentation, and microbial community in a rumen simulation (RUSITEC) system. The present results suggest that, compared to the other groups, rice straw with particle size of 4 mm may improve the disappearance rate of nutrients and promote the production of volatile fatty acids by regulating rumen microorganisms. Battelli, Colombini et al. tested bioactive compounds in two different *in vitro* studies. In the first study, the authors evaluated two additives, one with condensed tannins (CTs) from quebracho and one with hydrolysable tannins (HTs) from chestnut, at four inclusion levels (2%, 4%, 6%, and 8% on an as-fed basis) were added to the fermentation substrate and tested against a negative control. Both types of tannins significantly reduced the total gas (GP) and CH_4_ (ml/g DM) production during the 48 h of incubation. However, the lower levels of GP and CH_4_ production were associated with the reduction in dry matter digestibility caused by CTs and HTs. Conversely, no significant differences were observed for the protozoan and archaeal populations, suggesting a low direct effect of tannins on these rumen microorganisms *in vitro*. In the second study, Battelli, Nielsen et al. investigated the effects of catechin and quercetin (flavonoids), salicylic acid (phenolic acid), and tannic acid (hydrolysable tannin). The compounds were added to two different basal feed substrates (maize and grass silage) at three inclusion doses of 1.5%, 3%, and 6% of the feeds DM. This study demonstrated a dose-dependent ability of quercetin to reduce CH_4_ rumen emission, albeit the extent of CH_4_ suppression depended on the basal feed. Bezerra et al. studied the metabolism of crossbred Boer finishing goats fed diets containing crude glycerine from biodiesel production. Glycerine levels did not cause any adverse effects on the liver tissue, serum, or urinary profiles. The use of crude glycerine with a lower methanol content in goat diets is recommended. de Castro et al. evaluated the effects of including oilseed cakes on intake and digestibility, performance, carcass characteristics, and meat sensory properties in feedlot lambs. The inclusion of tucuma (*Astrocaryum aculeatum* M.) cake did not affect digestibility, but reduces intake and performance, and influence carcass characteristics and meat texture. Diets containing cupuassu (*Theobroma grandiflorum* K.) cake, or palmiste (*Roystonea oleracea* C.) cake reduced digestibility; however, intake, performance and carcass characteristics were similar to the control diet. Lata et al. structurally characterized two novel proanthocyanidins from *Anogeissus pendula* leaves. The novel proanthocyanidins have potential roles in improving feed conversion ratios and in drug development. He et al. investigated the effects of a mixed meal consisting of rapeseed meal, cottonseed meal, and sunflower meal in replacing soybean meal on growth performance, apparent nutrient digestibility, serum inflammatory factors and immunoglobulins, serum biochemical parameters, intestinal permeability, short-chain fatty acid content, and gut microbiota of finishing pigs. This study showed that the use of mixed meal as a substitute for soybean meal in the diet had no significant negative effects on the growth performance, nutrient apparent digestibility, serum immunoglobulins, serum antioxidant capacity, intestinal permeability, short-chain fatty acid content, and gut microbiota diversity in finishing pigs.

## Alternative feed in poultry

Regarding the use of alternative feeds in poultry nutrition, the researchers evaluated the use of feed and probiotics to improve animal health and performance of animals as an alternative to antibiotics. Attia et al. investigated the use of *Saccharomyces cerevisiae* and *Lactobacillus acidophilus*, with or without a prebiotic (mannooligosaccharide, MOS), as alternatives to zinc bacitracin (ZnB). The authors demonstrated that the addition of 2 g/kg of *S. cerevisiae* to broiler diet can effectively replace ZnB and improve production performance and economic return. Lefter et al. assessed the nutritional quality of cowpea seed (*Vigna unguiculata* L., cv. Doljana-CSD) and the impact of partially replacing soybean meal with CSD, along with the supplementation of microencapsulated *Lactobacillus salivarius (*LS), on the growth performance, selected carcass traits, plasma biochemical profile, tibia bone quality, and microbial populations in the ceca and fecal excreta of broiler chickens. This study suggests that cowpea seeds can be used as a partial replacement for soybean meal in broiler diets, and microencapsulated *Lactobacillus salivarius* can be used as a probiotic supplement. Huang et al. investigated the effects of Pu-erh tea pomace (PTP), a solid substance after extraction of functional substances, on the growth performance and gut microbes of chickens. The PTP could reduce the blood cholesterol levels by improving the composition of gut microbiota, providing a reference for the application of PTP in the poultry industry. Luo et al. tested *Litsea cubeba* (Lour.) Pers., a traditional Chinese herb with antibiotic-like properties, in broilers. The authors found that the extract had a positive effect on amino acid content and minor unsaturated fatty acids, thus improving flavor and nutritional value of the meat. These results suggest that *L. cubeba* extract, at any dose, could serve as a sustainable alternative to antibiotics, thus reducing the risk of drug resistance while improving meat quality, nutrition, and flavor. Sindaye et al. conducted a study to evaluate the effects of dietary lysozyme supplementation on laying hens' performance, egg quality, biochemical analysis, body immunity, and intestinal morphology. Dietary supplementation with lysozyme could improve intestinal morphology, immune efficiency, and nutrient digestibility in laying hens. In addition, a dietary supplement of 200 to 300 mg/kg lysozyme should be suggested to farmers as an appropriate level of feed additive in laying hen production. Spínola et al. emphasize the need for further research on optimal inclusion levels, processing methods and potential enzymatic enhancements of Spirulina in broiler diets. Cornescu et al. suggested that zinc-enriched yeast and parsley minimized the effects of heat stress on production performance parameters with a demonstrated role of antioxidant capacity by delaying the lipid peroxidation during different storage times. Paredes-Lopez et al., observed that *M. citrifolia* at 0.01% of the diet improved the intestinal health and thus the performance indices of the broiler chickens and did not have a detrimental effect on any of the parameters evaluated, postulating it as a potential alternative in poultry nutrition. According to Al-Harthi et al., *Moringa peregrina* seed meal (MPSM) can be included in the diet of broilers at a level of 10% level without negative effects on performance, carcass traits, meat quality, and blood lipids.

## Conclusion

In conclusion, the articles presented in this editorial demonstrate that alternative feeds and additives could be a useful tool to reduce the environmental impact of livestock animals without adverse effects on animal performance and products. Furthermore, alternative feed could improve animal health. However, further studies are needed to find the right dose to include these feeds in animal diets to avoid a reduction in degradability and adverse effects on animal performance compared to conventional feeds.

## Author contributions

AV: Writing – original draft, Writing – review & editing. FS: Writing – original draft, Writing – review & editing. DC: Writing – original draft, Writing – review & editing. IF: Writing – original draft, Writing – review & editing. AA: Writing – original draft, Writing – review & editing. MT: Writing – original draft, Writing – review & editing.

## References

[1] De Quelen F Brossard L Wilfart A Dourmad JY Garcia-Launay F. Eco-friendly feed formulation and on-farm feed production as ways to reduce the environmental impacts of pig production without consequences on animal performance. Front Veter Sci. (2021) 8:689012. 10.3389/fvets.2021.689012 34295934 PMC8289902

[2] Herrero M Henderson B Havlík P Thornton PK Conant RT Smith P. Greenhouse gas mitigation potentials in the livestock sector. Nat Clim Change. (2016) 6:452–461. 10.1038/nclimate2925

